# Comparing stem cells, transdifferentiation and brain organoids as tools for psychiatric research

**DOI:** 10.1038/s41398-024-02780-8

**Published:** 2024-02-28

**Authors:** Alfredo Bellon

**Affiliations:** 1 Penn State Hershey Medical Center, Department of Psychiatry and Behavioral Health, Hershey, PA USA; 2 Penn State Hershey Medical Center, Department of Pharmacology, Hershey, PA USA

**Keywords:** Psychiatric disorders, Stem cells

## Abstract

The inaccessibility of neurons coming directly from patients has hindered our understanding of mental illnesses at the cellular level. To overcome this obstacle, six different cellular approaches that carry the genetic vulnerability to psychiatric disorders are currently available: Olfactory Neuroepithelial Cells, Mesenchymal Stem Cells, Pluripotent Monocytes, Induced Pluripotent Stem Cells, Induced Neuronal cells and more recently Brain Organoids. Here we contrast advantages and disadvantages of each of these six cell-based methodologies. Neuronal-like cells derived from pluripotent monocytes are presented in more detail as this technique was recently used in psychiatry for the first time. Among the parameters used for comparison are; accessibility, need for reprograming, time to deliver differentiated cells, differentiation efficiency, reproducibility of results and cost. We provide a timeline on the discovery of these cell-based methodologies, but, our main goal is to assist researchers selecting which cellular approach is best suited for any given project. This manuscript also aims to help readers better interpret results from the published literature. With this goal in mind, we end our work with a discussion about the differences and similarities between cell-based techniques and postmortem research, the only currently available tools that allow the study of mental illness in neurons or neuronal-like cells coming directly from patients.

## Introduction

Despite intense research, the pathophysiology of psychiatric illnesses remains poorly understood. One of the major obstacles is the inaccessibility of neurons coming directly from patients. Several different approaches have been undertaken to gain access to neurons or neuronal-like cells coming directly from patients. These methodologies include: Olfactory Neuroepithelial cells, Mesenchymal Stem Cells, Pluripotent Monocytes, Induced Pluripotent Stem Cells, Induced Neuronal cells and more recently Brain Organoids. Each of these techniques has its own set of advantages and disadvantages which will be discussed in this manuscript. But before covering the specifics of each approach, it is important to address the main criticisms and confusions associated with the study of psychiatric illnesses at a cellular level.

The primary concern of working with cells in vitro consists of the inability to reproduce the living environment in which cells mature and interact. While this is a valid concern, the possibility of studying neuronal processes directly in cells carrying the genetic predisposition to the illness in question is invaluable and as of yet, inaccessible through any other means. Moreover, most psychiatric illnesses are polygenic disorders with a growing number of genes linked to their pathophysiology. Several of these genes orchestrate processes that point to deficits at the cellular level [1–4]. Thus, studying cells in vitro that can reproduce specific neuronal processes suspected to be compromised in psychiatric illnesses is a necessary step in our quest to understand such ailments.

There has been significant confusion about what cellular approaches can “model” when studying psychiatric disorders. Since the pathophysiology of these diseases is not known, let alone at a cellular level, cells in vitro cannot be considered “models” of psychiatric illnesses under any circumstances, at least at present time. Even cellular aspects of normal brain physiology continue to be debated. What cellular approaches can do, is replicate at least some neuronal processes in vitro that can be later contrasted in case-control studies. The value of each cellular approach depends on how precisely and consistently the neuronal process in question can be replicated.

None of the methods currently available delivers actual neurons with the exception of biopsies from the olfactory epithelium [5]. Nonetheless, the prospect of manipulating and challenging live neuronal-like cells in vitro that carry the genetic predisposition for psychiatric illnesses provides a unique research avenue. Moreover, neuronal-like cells that come directly from patients have the potential of becoming or help identify biomarkers. This is in contrast with other valuable research approaches such as the use of postmortem tissue or animal models, which do not offer this possibility. In fact, much of the enthusiasm surrounding the discovery of Induced Pluripotent Stem Cells resulted from the potential for these cells to influence patient care. While this proposition has not yet been accomplished, likely due to deficiencies in results reproducibility [6–9] as well as the long time it takes for somatic cells to deliver differentiated neuronal-like cells [10], we have to keep in mind that access to living neuronal-like cells in vitro coming directly from psychiatric patients is a relatively recent event that started inconspicuously with biopsies from the olfactory epithelium in 1998 [11] and exploded around 2016 a decade after the discovery of Induced Pluripotent Stem Cells [12]. To place this in perspective, the field of neuroimaging and schizophrenia started in 1976 with the landmark work by Johnstone et al. [13] and rapidly expanded shortly after.

There is no question that significant work remains within the field of cellular approaches to psychiatric illnesses. But considering the complexity of the human brain and the difficulty of accessing neurons, our righteously dampened expectations on the potential clinical applications of neuronal-like cells coming directly from patients, should not be completely extinguished. Instead, what is essential to foster further progress in this field, is to understand each of the methodologies available in detail. In order to contribute to this goal and to allow researchers to determine which approach is better suited to recapitulate the human disease in question and, at the same time, be practical within the limits of the available technology, here we contrast advantages and disadvantages of each cellular approach currently in use, placing particular emphasis on non-genetic transdifferentiation of human monocytes into neuronal-like cells. We also describe how transdifferentiated human monocytes are presently used in neuroscientific research including our recent results studying schizophrenia at a cellular level.

### Embryonic stem cells

**Fig. 1:**
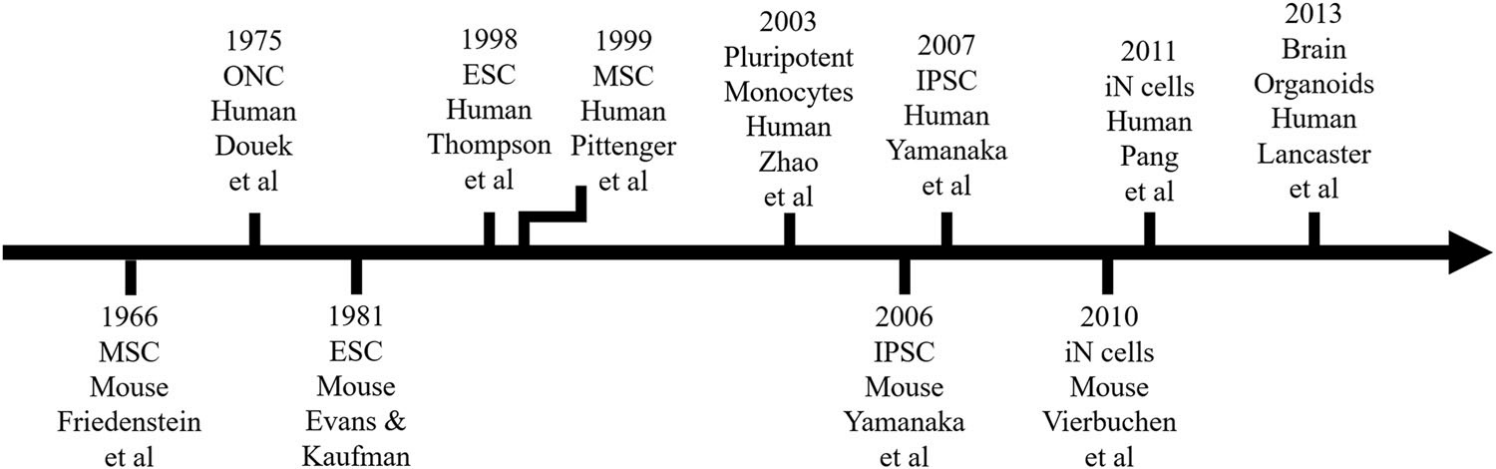
Timeline on the discovery of stem cells and other cellular research tools used in psychiatry.

Embryonic Stem Cells (ESCs) were first isolated from human blastocysts in 1998 [14] several years after their discovery in mice [15] (Fig. 1). ESCs are the gold standard in stem cell biology due to its ability to continuously replicate and most importantly differentiate into cells from any of the three germ layers [16]. ESCs can be maintained in vitro for months before differentiation into neuronal-like cells with immature as well as mature characteristics [17]. Not surprisingly, protocols to differentiate a wide variety of neuronal types have been described [18–20]. But researchers working with ESCs face many hurdles.

Isolation and culture of ESCs is technically challenging despite the many methodologies currently in practice [21]. More significant than the technical difficulties, are the ethical implications of harvesting and discarding human embryos for research purposes [22]. Moreover, ESCs cannot be used for case-control studies, as these cells do not originate from identifiable patients. In terms of treatment applications, the original expectations that ESCs could revolutionized regenerative medicine has been curtailed due to the immune reaction these cells can provoke [23] and because of the risk to trigger the formation of teratomas [24]. As of 2018, only two clinical trials reported successful results when using ESCs [25]. Instead, the role of ESCs within the field of cellular approaches to psychiatric illnesses has been as the gold standard for differentiation and cell replication in relation to other stem cell types [26].

### Mesenchymal stem cells

Mesenchymal Stem Cells (MSCs) are adult stem cells characterized by the expression of a specific group of surface markers [27]. MSCs were first isolated from the bone marrow of mice in 1966 [28]. It appears that these stem cells did not generate significant interest as no studies were undertaken in humans for decades. It was not until ESCs were discovered that MSCs were finally harvested from humans [29], over thirty years after its isolation from mice (Fig. 1). Now it is known that MSCs can be isolated from a variety of different tissues such as dental pulp and skin, though their most common source for clinical and research purposes continues to be the bone marrow [30]. This type of adult stem cells can be differentiated into neuronal lineage in 6 to 14 days [31, 32]. The process of differentiation starts when MSCs reach confluency, which occurs at least 10 days after bone marrow aspirates take place [29, 33]. MSCs replicate rapidly and therefore, can easily be expanded [29, 33]. Another advantage offered by MSCs is that multiple protocols are already available to differentiate these stem cells into various neuronal types [34] some of which are mature enough to conduct electrical activity [35]. The majority of these protocols rely on adding growth factors and other chemical components to the culture media without the need for viral insertion into the cell’s genome [34] (Table 1).

**Table 1:** Cellular approaches used in psychiatry.

Cellular Approach	Access	Requires Reprograming	Ability to Replicate	Time to deliver differentiated cells*	Homogeneous Differentiation	Differentiation Efficiency	Degree of differentiation	Reproducibility of results	Cell dead after differentiation	Cost
Mesenchymal Stem Cells	Invasive	No	Yes	16–24 days	No	Medium to High	High	Not studied	High	Medium^**^
Olfactory Neuroepithelial Cells	Invasive	No	Yes	Days to 4 weeks	No	Medium to High	Low^#^	Low^##^	Not studied	Medium^**^
Induced Pluripotent Stem Cells	Invasive^+^	Yes	Yes	4–6 months	Yes, with some protocols	High	High	Low	Low	Medium to High
Induced Neuronal Cells	Invasive^+^	Yes/No^++^	No	2–5 weeks	Yes, with some protocols	Medium to High	High	Low	Low	Medium to High
Brain Organoids	Invasive^+^	Yes	Yes	4–6 months	Yes, with some protocols	High	Medium	Medium^+++^	High	High
Pluripotent Monocytes	Non-invasive	No	Limited	5–30 days	No	Low	Low	Medium^^^	High	Low

*From somatic to neuronal-like cell. **Requires consulting a specialist. ^#^When working with neuroprogenitor cells. ^##^Due to variability between biopsies. ^+^Most protocols originate from skin fibroblasts. ^++^No when small molecules are exclusively used. ^+++^For Forebrain Organoids. ^^^Needs replication by an independent team.

Accessibility is the main obstacle posed by MSCs, as bone marrow aspirates from the superior iliac crest of the pelvis continue to be the most common harvesting method [33]. This is an invasive procedure that requires local anesthesia and could lead to medical complications [36]. Another disadvantage is that MSCs’ response to neuronal differentiation is heterogeneous [31, 32, 37] and high differentiation efficiency leads to increased cell dead [38]. Moreover, reports indicating whether or not MSCs deliver reproducible results with serial samples from the same individual are still lacking.

MSCs are not currently used to investigate potential pathophysiological mechanisms in psychiatry. This is likely because there are other less invasive methods to obtain neuronal-like cells directly from patients. Instead, MSCs are being tested as potential treatment alternatives [39]. MSCs express low levels of major histocompatibility complex class I molecules and do not express major histocompatibility complex class II [39]. This pattern of expression of surface markers significantly reduces the potential for MSCs to elicit immunological reactions after allogenic transplants. Its safety has been documented in at least two meta-analyses comprising more than 80 clinical trials [40, 41]. The hypothetical benefit of MSCs relied originally on the possibility of stem cells to replace ailing somatic cells and more recently, on its immunomodulatory properties capable of reducing inflammation [42]. Its treatment potential has been tested in a wide variety of illnesses raging from cardiovascular diseases to neurological disorders such as Parkinson’s disease, stroke and traumatic brain injury [16, 34, 40, 41]. Within the psychiatric field, the focus has been on autism. Currently, there are several ongoing clinical trials in many countries including the US, assessing the clinical impact of MSCs in children with autism spectrum disorder [39, 43]. While the safety of MSCs has been well established, their therapeutic value remains to be proven [39]. Evidence about the therapeutic potential of MSCs for illnesses such as Parkinson’s disease, Amyotrophic lateral sclerosis and autism spectrum disorder ranges from weak to non-existent [39, 44, 45]. Particularly lacking is data about the long term clinical consequences of MSCs therapy.

### Olfactory neuroepithelial cells

The olfactory epithelium’s capacity to regenerate allows researchers to access neurons at various stages of differentiation ranging from neuroprogenitor cells to mature olfactory sensory neurons [5, 11, 46]. This variety of olfactory neuroepithelial cells (ONCs) can be studied through two main techniques: cellular and histological. The cellular approach involves either, generating neurospheres based on the mitotic capacity of neuroprogenitor cells, or by directly analyzing adherent dissociated cells omitting sphere formation [5, 46]. The histological method consists of culturing intact olfactory tissue, which maintains cells in a more physiological environment [5]. This ex vivo paradigm contains stratified stages of neuronal maturation, leading some authors to suggest that it provides a window into human neurodevelopment [5, 11]. Granted, the histological method’s experimental versatility is limited when compared with dissociated cells in vitro [46].

The diversity of cells obtained from the olfactory epithelium is not limited to neurons at different stages of maturation. Epithelial and glial cells are also present [5] which could be a hindrance when the goal is to generate homogenous cell cultures. In order to produce more homogeneous cell cultures, it is necessary to generate neurospheres using neuroprogenitor cells [47]. This approach also offers the advantage of yielding large number of replicating cells that are ready for experimentation after approximately 4 weeks [47]. A faster method to obtain ONCs in vitro is to work with dissociated cells that adhere to the culture surface shortly after extraction. However, such technique circumvents cell propagation and thus limits the number of cells available for research. This timeline from extraction to experimentation ranging from a few days to 4 weeks is one of the fastest currently available (Table 1). An additional benefit is that ONCs do not require reprogramming. Reprograming entails reverting somatic cells into less differentiated stages so that these somatic cells acquire stem cells properties [48]. Some cellular approaches to psychiatric illnesses depend on this reprograming process as will be discussed later in this manuscript.

While ONCs offer access to mature neurons coming directly from patients, these are olfactory sensory neurons [11], which contribution to the pathophysiology of psychiatric illnesses is limited. However, ONCs also provide neuroprogenitor cells that genetically resemble other stem cells [49] but with the advantage of expressing numerous neuronal-specific genes [50]. In addition, recent single-cell RNA results indicate that neuroprogenitor cells obtained from the olfactory epithelium, closely match embryonic brain cells [51]. If these latter results are confirmed, ONCs would not only be a source of stem cells, instead, they could also provide direct access to early neuronal development without the need for any genetic manipulation. Moreover, such cells could be differentiated into any disease relevant neuronal type and thus, could have profound implications for the study of neurodevelopmental disorders like autism and schizophrenia. Further research in this area though, is still needed.

Access to ONCs requires a biopsy of the olfactory mucosa, which was first described in 1975 [52] (Fig. 1) and then improved in 1982 [53]. Its use in psychiatry as a source of live cells did not start until 1999 [54]. A recently developed less invasive approach relies on cell exfoliation instead of biopsying the olfactory epithelium [55] but both procedures should be performed under local anesthesia by a qualified otorhinolaryngologist [5, 11]. Therefore, the main disadvantage of working with ONCs is accessibility (Table 1). In addition, the need for a medical specialist increases the cost for this cellular approach. There are also reports indicating that results could vary between biopsies from the same individual [5, 11]. Lastly, the capacity for neurosphere-derived olfactory neuronal-like cells to conduct electrical activity remains under investigation therefore, the degree of maturity that can be reached with these neuroprogenitor cells is currently limited [5, 55].

### Induced pluripotent stem cells & brain organoids

The unexpected possibility of inducing pluripotency in already differentiated somatic cells [12], relaunched and is now, leading the field of cellular approaches to psychiatric illnesses (for a review refer to McNeill et al. [56] & Unterholzner et al. [57]). In its conception, this monumental discovery relied on skin fibroblast as the somatic cell of origin [12, 58, 59]. Now, IPSCs can be generated from a variety of cell types including blood cells [60] and keratinocytes [61] which allow for easier access. But the main advantage offered by IPSCs is not accessibility. It is the possibility of generating a wide variety of neuronal types carrying a meticulous degree of specification [62]. For instance, researchers can induce differentiation into hippocampal dentate granule cells or midbrain dopaminergic neurons among many others [63]. Neuronal-like cells generated from IPSCs are the closest to neurons that researchers can get when relying on differentiating protocols. Due to its in vitro origin and a perpetual state of maturation however, these cells should not be considered neurons. While this immature stage limits IPSCs-generated-neurons for the study of neurodegenerative diseases, it is advantageous when approaching illnesses with neurodevelopmental origins such as autism and schizophrenia [10].

Induced pluripotency is achieved by reprograming the somatic cell’s genome [12]. Originally, the reprograming process depended on retroviruses for integration into the cell’s DNA of four transcription factors *Klf4*, *Oct4*, *Sox2* and *c-Myc* [12]. Since then, other integrative and non-integrative approaches have emerged. Shortly after the discovery of IPSCs, retroviruses were replaced by lentiviruses [64]. But because of the potential for these integrative methodologies to elicit genetic and epigenetic abnormalities [10, 26, 65–70] other non-integrative strategies have been developed such as; Sendai viruses [71], plasmids [72], modified RNA [73] and small molecules [74, 75]. All of these reprograming methods have its own set of advantages and disadvantages which have been reviewed elsewhere [76, 77]. But currently, Sendai viruses have become the preferred reprograming method [77]. Regardless of which reprograming protocol is followed, somatic cells are dedifferentiated, and with that, replication resurges giving researchers the possibility to expand induced-pluripotent cells (Table 1). These expanded IPSCs are known as cell lines that originate from a single somatic cell [10]. Then, the wide variety of differentiation protocols now available can be put into practice [62]. Researcher can expect higher differentiation efficiency with some of the most recently developed protocols [78]. Unfortunately, not all IPSCs respond equally and consequently, the resulting cell cultures are often heterogeneous (Table 1). For some neuronal types however, there are differentiating protocols that yield high levels of homogeneity [78].

In many aspects, IPSCs technology has advanced rapidly and this progress has positively impacted psychiatric research at the cellular level. Historically, IPSCs have been used in schizophrenia and bipolar disorder [56], but there are now IPSC lines that allow researchers to study alcohol use disorder [79], cannabis use disorder [80], opioids use disorder [81] and the effects of selective serotonin reuptake inhibitors (SSRIs) [82]. The versatility that IPSCs offer is also evident at the genetic level. Cell lines carrying exonic deletions [83], de novo mutations [84], missense mutations [85] and copy number variants (CNV) [86] among many others genetic abnormalities have been created. Another recently discovered genetic approach is entering the IPSCs field. The gene editing tool CRISPR-Cas9, is being used to create isogenic IPSCs, either to introduce disease-causing mutations or to remove them [87]. The application of CRISPR-Cas9 to IPSCs is helping investigate the pathophysiology of schizophrenia [88, 89] and other psychiatric disorders [81]. One more area in which IPSCs are breaking ground is drug discovery. Patient-derived cell lines are being tested as predictors of pharmaceutical toxicity, for drug target validation and as screening tools for already clinically approved or experimental compounds [90]. There are however, just as with any other research tool, challenges when generating IPSCs.

One of the limitations of working with IPSCs is the time needed from acquisition of somatic cells to differentiated neuronal-like cells. This gap takes between 4-6 months [7, 10]. Such a long period of time, constraints researcher to study only a small number of individuals simultaneously. Though, the advent of non-integrative approaches is shortening the reprograming process [77]. Another disadvantage is that the reprogramming process elicits genetic and epigenetic anomalies [26, 65–70] that can become confounders when studying polygenic illnesses with poorly understood genetic loads like most psychiatric disorders. But perhaps the main obstacle when working with IPSCs is heterogeneity between cell lines from the same individual [6–9]. This variability impedes our ability to draw firm conclusions from case-control studies. In order to circumvent this hurdle, researchers are studying illnesses with clearly identifiable genetic abnormalities [91, 92] which allow for clear comparisons between IPSCs carrying the genetic anomaly and cell lines from controls free of such defect.

Currently, the most significant advance in stem cell biology is the creation of organoids, which are most commonly created by combining IPSCs and ESCs. Taking advantage of new technology in cell culture and the capacity of cells to self-organize, Sato et al. developed in 2009 the first three-dimensional (3D) in vitro culture of a tissue, specifically; intestinal epithelium [93]. Building from this monumental finding, Lancaster and colleagues developed in 2013 the first Brain Organoids [94]. Brain Organoids are formed by organized groups of interacting neurons and glia at different stages of development [95–97]. Neurogenesis, gliogenesis and synaptogenesis can be found in these complex 3D cultures [95–97]. Even cavities resembling immature ventricles are encountered [95–97]. Several aspects of neurodevelopment can be studied within Brain Organoids such as neuronal migration [96] and neuronal maturation involving different types of neurons and glia [95–97]. This technology is advancing rapidly. Now a days, there are protocols available to generate Brain-region-specific Organoids such as forebrain, midbrain, hypothalamus, hippocampus, basal ganglia and cerebellum [95]. Fusion of two different Brain-region-specific Organoids is also possible. The recent fusion of Thalamic Organoids with cortical ones provided insights into axon targeting and synaptogenesis [98]. Not surprisingly, Brain Organoids have already been used to improve our understanding of psychiatric illnesses (for a review refer to Whiteley et al. [95] & Unterholzner et al. [57]).

As with any other research technique, Brain Organoids have also disadvantages. While neurons within brain organoids can be electrically active [99], neuronal maturation is impeded by necrosis at the center of the organoid resulting from longer culture times [97, 100]. Brain Organoids are not vascularized therefore, the amount of nutrients cells at the core receive decline as the culture progresses [97, 100]. In addition, Brain Organoids remain small when compared to human brains and its shape lack the dorsal-ventral axes organization that is seen under physiological conditions [100]. The time and equipment needed to generate brain organoids is also problematic. Special equipment beyond that encountered in standard tissue culture laboratories is required, such as particular types of incubators and oxygen lines [100]. In terms of time, we have to consider that the gap involving isolation of fibroblasts, genetic transduction, IPSC line expansion and selection is 4–5 months [7, 10] and the culture time from IPSCs to Brain Organoid is approximately 40 days [100]. Making Brain Organoids is therefore a lengthy process that requires a high level of expertise [100]. In addition, the most common protocols used to generate IPSCs come from skin fibroblasts and as mentioned in previous paragraphs, this requires a skin biopsy, an invasive procedure performed by a medical specialist [101]. But perhaps the most concerning limitation is the degree of inter and intra individual heterogeneity yielded by Brain Organoids [100, 102, 103], though in some areas, such as cell population in Forebrain Organoids, ground has been gained [104].

### Induced neuronal cells

Induced Neuronal (iN) cells were created following the same rationale that led to the discovery of IPSCs, namely, manipulating the cell’s genome [105, 106]. For iN cells however, the goal is to force expression of transcription factors that directly generate neurons avoiding dedifferentiation into pluripotent stem cells [107]. This saves time (Table 1). Direct genetic-transdifferentiation from somatic cell to iN cells takes between 4–5 weeks when using transcription factors [106]. The differentiation time can be reduced to 14 days if small molecules are used [108]. This faster approach however, leads to heterogeneity in cell maturation [108]. In addition to being a faster method to generate neuronal-like cells, iN cells offer another advantage over IPSCs. Induced Neuronal cells do not originate from cloning one single cell [10]. Instead, many somatic cells of origin, most commonly skin fibroblasts [10, 107], are simultaneously exposed to direct lineage reprogramming [10]. This cellular diversity is better equipped to deliver generalizable results [10]. In addition, by avoiding the reprogramming process that leads to pluripotency, iN cells are able to maintain the epigenetic age of the cell of origin as there is no dedifferentiation into more primitive cellular stages [109]. By preserving its epigenetic age, iN cells are better suited for the study of psychiatric disorders with onset in or after adulthood [10], while, its usefulness for understanding illnesses with neurodevelopmental origins is limited [10, 110] One aspect of iN cells that has received less attention, is whether these cells DNA methylation patterns resemble those of the neurons they are meant to mimic. There are some indications that they do. Induced Neuronal cells from mice recapitulate DNA methylation patterns found in postmitotic neurons [111]. Nonetheless, similar work using human iN cells is lacking.

Some of the limitations encountered in IPSCs are also present in iN cells (Table 1). For both methods, the most widely used cell of origin is skin fibroblasts [10, 64, 109]. In order to access skin fibroblasts a skin biopsy is needed which implicates a minor surgical procedure performed under local anesthesia in consultation with a specialist [101]. For IPSCs, many other cells of origin have been tested [64]. But for iN cells the alternatives are still limited [109]. Therefore, access to iN cells is laborious and invasive (Table 1). Similar to IPSCs, concerns have been raised about the reproducibility of results obtained with iN cells [109] putting in question its potential application for clinical studies. There is hope however, that because iN cells can be generated more rapidly and consequently, more individuals can be studied simultaneously, either a statistical or a bioinformatic solution could be found in the near future [109]. Another down side is that iN cells cannot be expanded (Table 1) as the resulting neurons are postmitotic [107]. Moreover, the type of neurons yielded is often heterogeneous, though progress has been made in this area [112]. Finally, iN cells generated exclusively by the addition of small molecules [113] as well as other non-genetic transdifferentiation methods like the use of pluripotent monocytes [114] could, in theory, avoid the formation of genetic and epigenetic abnormalities elicited by the reprograming process. Though, further research is needed in this direction.

### Human circulating pluripotent monocytes

Two independent teams showed in 2003 that a subpopulation of human circulating monocytes has pluripotent capabilities [115, 116]. Their hypothesis originated from the physiological ability of monocytes to differentiate into various types of phagocytic cells [117–120], as wells as, reports indicating that under certain culture conditions, monocytes could become endothelial-like cells [121, 122]. Both teams identified this subset of monocytes as cells expressing CD14, CD34 and CD45 [115, 116]. CD14 is a marker for monocytes while, CD34 and CD45 are markers of hematopoietic stem cells. Expression of CD34 in monocytes was identified via flow cytometry or fluorescence microcopy after 5–7 days in culture [115, 116]. We also found expression of CD34 in monocytes after 7 days in culture following our transdifferentiation protocol [114]. An Italian laboratory using high sensitive flow cytometry took these results a step further. Romagnani and colleagues, demonstrated that 5–10% of circulating CD14+ cells express low amounts of CD34 [123] which indicates that expression of CD34 is inherent to a subset of circulating monocytes and thus, its independent of cell culture conditions. Pluripotent monocytes, in contrast to circulating hematopoietic stem cells [124], do not originate from cells expressing CD34. On the other hand, the difference between endothelial progenitor cells (EPC) and pluripotent monocytes is that these latter cells originate from circulating CD14+ cells, while the surface markers that characterize EPC are Vascular Endothelial Growth Factor Receptor 2 (also known as KDR), CD133+ and CD34+ [125].

Pluripotent monocytes also carry other characteristics of stem cells such as the potential to replicate. While one team did not [126], several independent laboratories, including ours, encountered that a subpopulation of monocytes can proliferate under certain culture conditions. [114–116, 123, 127] We only found a small degree of replication, which in our hands, limits the expandability of pluripotent monocytes [114]. In contrast, Zhao and colleagues were able to expand and store these cells for future experiments [115]. Results from Mishra et al. suggest replication in pluripotent monocytes is only temporary, peaking at day 6 and abating by day 10 [127]. These discrepancies in replication rates could be explained by methodological differences in generating pluripotent monocytes.

There are several protocols currently available to elicit pluripotency in monocytes. The common denominator is that these protocols do not require reprograming or any type of genetic manipulation. Instead, non-genetic-transdifferentiation is achieved by adding growth factors to the culture media and more often than not, by coating the cell culture surface with human fibronectin. Zhao et al., one of the original teams that reported pluripotency in monocytes, relied on macrophage colony-stimulating growth factor (MCSF) but no coating [115] whereas, Kuwana and colleagues, the other laboratory to first report pluripotency in monocytes, coated cell culture plates with human fibronectin but did not use MCSF [116]. Kuwana et al. later showed that coating culture plates with human fibronectin [128] and the presence of Stromal Cell-derived Factor-1 (SDF-1) either from peripheral blood mononuclear cells (PBMCs) or commercially available SDF-1 [129] are necessary for monocytes to acquire pluripotency. In our experience, coating cell plates with fibronectin is indispensable. We also use MCSF and PBMCs conditioned media to elicit pluripotency in monocytes [114]. Other protocols that prompt stem cell-like behavior in monocytes have also been published [123, 126, 127, 130]. All of these approaches use standard cell culture techniques and basic laboratory equipment making non-genetic-transdifferentiation of monocytes an accessible and inexpensive (Table 1) alternative to obtain neuronal-like cells directly from patients. The disadvantages of generating neuronal-like cells via non-genetic-transdifferentiation of monocytes will be described in the following paragraphs.

### Non-genetic-transdifferentiation of human monocytes into neuronal-like cells

Together, the two reports that demonstrated monocytes pluripotent capacities for the first time, transdifferentiated these cells into neurons, lymphocytes, hepatocytes, osteoblasts, myoblasts, chondrocytes, adipocytes, epithelial and endothelial cells [115, 116]. Their characterization however, consisted almost exclusively on the expression of markers for each cellular lineage. Since then, many other teams have replicated their findings and expanded the characterization of differentiated cells [123, 130, 131]. Protocols to generate other cell types such as pancreatic islet cells with the capacity to synthetize insulin have also been created [132]. In addition, a better characterization of the neuronal-like cells obtained from pluripotent monocytes is now available.

In 2006, Kuwana and colleagues, published a report specifically about monocytes differentiation into neuronal-like cells [133]. Their protocol consisted of co-culturing embryonic rat neurons with what they referred to as Monocyte-derived multipotential cells (MOMC). Three days in co-culture led to the expression of early neuroectodermal transcription factors including Mash1, Ngn2 and NeuroD. After day 3, nestin was detected. Two weeks later, the resulting neuronal-like cells acquired unipolar, bipolar and multipolar shapes and expressed a variety of neuronal markers, such as low-molecular weight neurofilament, NeuroD, Hu, NeuN, MAP2, β3-tubulin and GAP43. During the transdifferentiation process many cells detached and died. The entire protocol took 24 days including MOMC generation which lasted between 7–10 days [133].

In 2010, a German team tested whether monocytes expressing CD34 and CD105 (a mesenchymal marker) could be differentiated into dopaminergic neuronal-like cells [126]. About 50% of cells positive for CD34 and CD105 also expressed nestin and some cells showed weak immunofluorescence to Tau and β3-tubulin. Treatment with sonic hedgehog (SHH) together with epidermal growth factor (EGF) and fibroblast growth factor 8b (FGF8b) for 14 days followed by another 14 days in culture without those compounds, elicited cells with neuronal shape and strong immunofluorescence to Tau and β3-tubulin, while nestin was no longer present. Ninety percent of neuronal-like cells expressed MAP2 and the dopamine transporter (DAT). Such proteins were not evident in undifferentiated cells. This protocol required 5 days to elicit the expression of CD34 and CD105 in monocytes plus 28 days for neuronal induction [126].

A year after the German team tested the potential for pluripotent monocytes to deliver dopaminergic neuronal-like cells, a laboratory from China reported that pluripotent monocytes can also be transdifferentiated into retinal-like cells [134]. Liu and colleagues cultured PBMCs either with neural stem cell medium or with retinal conditioned media. This latter culture media was prepared by exposing neural stem cell medium for three days to retinal tissue extracted from new born Sprague-Dawley rats. After five days in culture with either media, cells exhibited neuronal-like and glial-like shape. Nestin, MAP2, and glial fibrillary acidic protein (GFAP) were found in cells coming from both groups. CD16, an immunologic marker, was highly expressed in cells treated with neural stem cell medium, while cells cultured with retinal conditioned media evidenced vimentin as well as a specific retinal protein; rhodopsin. More recent projects, have shown that these retina-like cells also express β3-tubulin [135, 136].

In a current project developed by a New Delhi based laboratory, human circulating monocytes were transdifferentiated into neuronal-like cells with features of retinal neurons [127]. They followed the protocol described by Ruhnke et al. to induce pluripotency in monocytes [130]. Treatment with MCSF, interleukin-3 (IL-3) and β-mercaptoethanol for 6 days resulted in down regulation of CD14 and a 25% increase in CD117 also known as stem cell growth factor receptor. These pluripotent monocytes also expressed CD34 and had the capacity to replicate [127]. Following, neuronal differentiation was pursued by supplementing culture media for 8 days with basic-FGF, retinoic acid, EGF, insulin growth factor-1 (IGF-1), stem cell factor (SCF), B27 supplement and ESCs serum. The resulting cells exhibited a variety of morphologies including neuronal-like shape and viability of around 40%. Over 80% of these retinal neuron-like cells (RNLCs) expressed paired box protein-6 (PAX-6) a retinal marker. Other retinal proteins like rhodopsin and recoverin were also present, but the exact percentage was not specified. Some of these markers were slightly evident in pluripotent monocytes but not in uninduced monocytes. Mishra et al. also showed that RNLCs changed its membrane potential when exposed to alternating light and dark conditions [127].

**Fig. 2:**
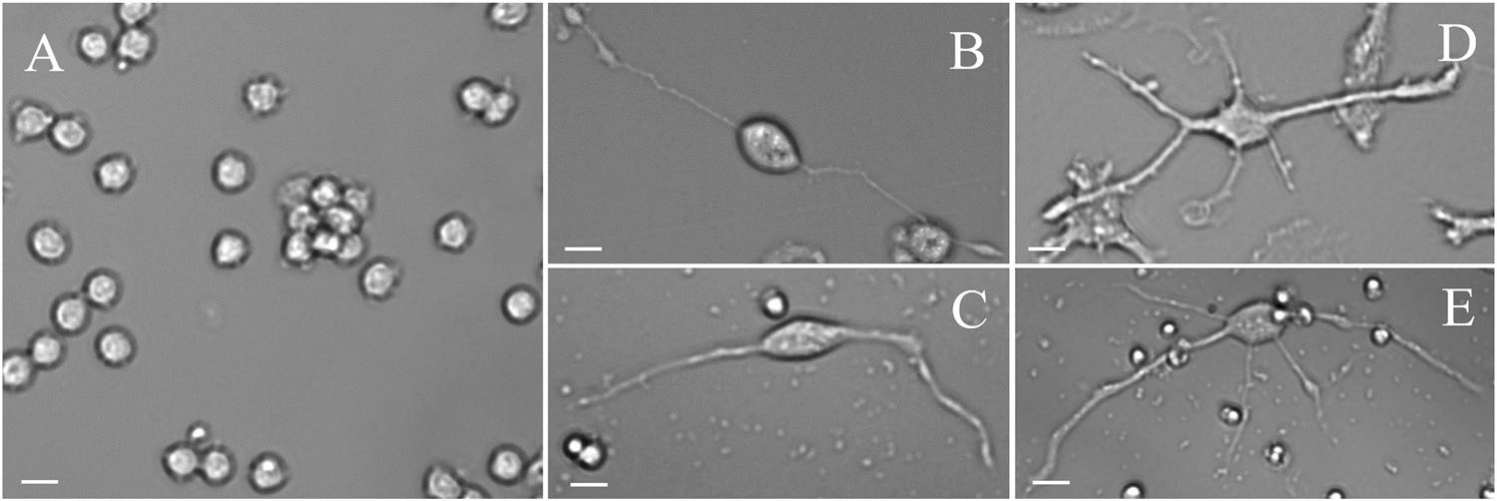
Light microscopy photographs. **A** Human monocytes shortly after isolation from blood. **B** Monocyte-Derived-Neuronal-like cell (MDNC) displaying bipolar shape. **C** Human Developing Neuron (HDN) after 5 days in culture displaying bipolar shape. **D** MDNC displaying multipolar shape. **E** HDN after 5 days in culture displaying multipolar shape. Magnification 20×. Scale bar = 20 µm.

Since circulating monocytes can be easily accessible via a standard blood sample and there is evidence from five independent research teams all located in different countries, that pluripotent circulating monocytes can be differentiated into neuronal-like cells, we set to develop a protocol that would deliver consistent and reproducible results to be used in the study of psychiatric and neurological illnesses. We also aimed to fill some literature gaps about the characterization of neuronal-like cells derived from monocytes. Our patented protocol, elicits pluripotency in monocytes by culturing these cells in fibronectin coated plates and supplementing media with MCSF [114]. We also use PBMCs conditioned media. Monocytes cultured following our protocol, express CD34 starting at day 7 and until day 10. On day 10 of culture, we start the transdifferentiation into neuronal-like cells by delivering the first dose of retinoic acid. On day 13, a second dose is applied together with butylated hydroxyanisole (BHA), IGF-1 and neurotrophin-3 (NT-3). Cells are then treated with potassium chloride (KCl) on day 17. By day 20 to 22, cells with neuronal shape are evident. These cells have a well-defined soma, which is either rounded or oval and thin long neurites, some of them longer than two times the soma size (Fig. 2). We named these cells Monocyte-Derived-Neuronal-like Cells (MDNCs). MDNCs decrease and in some instances completely abolish its expression of CD14 [114]. Other proteins present in monocytes such as CD11B and CCR2 are also absent in MDNCs [137]. Instead, these cells express a variety of neuronal markers. We have studied, through flow cytometry, immunofluorescence, RT-PCR and single cell mRNA sequencing either in combination or each technique individually (Table 2), the expression of 43 neuronal markers involved in synaptic functioning, neuronal structure [137] or considered as markers of specific neuronal types such as glutamatergic, GABAergic, dopaminergic, serotoninergic, cholinergic or motor neurons [114]. Of the 43 neuronal markers searched, MDNCs express 28 (Table 2). Several of these proteins are commonly found in mature neurons, namely, NeuN and MAP2 but others, like nestin, are found in developing neurons. We did not find expression of the glial marker GFAP [114]. When trying to determine if MDNCs belong to a specific neuronal type, it becomes evident that these cells are not committed into any specific neuronal lineage [114]. Instead, MDNCs express markers of several different neuronal types, such as glutamatergic, GABAergic, dopaminergic, serotoninergic and cholinergic but not motor neurons [114] (Table 2). This is likely because MDNCs remain in a developing stage that structurally resembles that of human neurons after 5 days in culture [114] (Fig. 2). A direct comparison between the structure of human neuroblastoma cells (NBCs), human developing neurons (HDNs) after 5 days in culture and MDNCs, revealed that MDNCs and HDNs extend primary neurites of close to 100 µm in average, while NBCs primary extensions were less than 80 µm [114]. The length of secondary neurites was comparable between the three cellular types, all reaching around 20 µm. In contrast, the number of primary and secondary neurites was higher in MDNCs. HDNs and NBCs extended about 3.5 primary neurites and between 1 to 2.8 secondary neurites. In order to determine how MDNCs acquire its neuronal shape, we followed five cells from two individuals via serial microphotographs [114]. We observed a similar structural transition as the one described for developing neurons in vitro [138].

**Table 2:** Neuronal markers expressed in Monocyte-Derived-Neuronal-like Cells (MDNCs).

	Neuronal Marker	Flow Cytometry	Immuno fluorescence	RT-PCR	Single cell mRNA sequencing
1	Neurofilament		present		
2	Nestin	present	present	present	
3	MAP-2		present	present	
4	β3-tubulin	absent		present	
5	PSD-95	present			
6	GAD-67	present		present	present
7	NeuN				present
8	BLOC1S3				present
9	vGLUT1				absent
10	vGLUT2				absent
11	NMDAR1				present
12	NMDAR2B				absent
13	Glutaminase				present
14	GABA transporter 1				absent
15	GABA type B receptor subunit 1				absent
16	GABA type B receptor subunit 2				absent
17	GAD-65				absent
18	Tyrosine hydroxylase				absent
19	DAT				absent
20	FOXA2				absent
21	Girk-2				present
22	Nurr-1				present
23	LMX1B				present
24	Tryptophan hydroxylase-1				present
25	Tryptophan hydroxylase-2				present
26	Serotonin transporter				present
27	PET-1				absent
28	Cholinacetyltransferase				absent
29	VAChT				absent
30	Acetylcholinesterase				present
31	Insulin enhancer protein				absent
32	Motor neurons and pancreas homeobox-1				absent
33	Neurexin-3				present
34	Synaptosome associated protein-25				present
35	Synaptic vesicle glycoprotein-2A				present
36	Vesicle associated membrane protein-1				present
37	SHANK-2				present
38	Synuclein alpha				present
39	Syntaxin-1A				present
40	SPIRE-1				present
41	Tau				present
42	Growth associated protein-43				present
43	GABA type A receptor subunit β-3				present

To further characterize the structure of MDNCs, we treated them with colchicine and dopamine. Colchicine can elicit neurite retraction in neurons [139] and neuronal cell lines [140] through microtubule depolymerization [141]. MDNCs retracted its longest primary neurite (LPN) and decreased the number of secondary neurites after treatment with colchicine [114] at a similar concentration and incubation time as those used for neurons and neuronal cell lines [139, 140]. Dopamine can also lead to neurite retraction in vitro [142–144] and in vivo [145]. Dopamine elicited retraction of MDNCs’ LPN and also reduced the number of secondary neurites. Pruning of LPN in MDNCs was elicited in part by activation of the dopamine 1 receptor [146] resembling what has been described in developing neurons [145]. Neither dopamine nor colchicine affected MDNCs’ longest secondary neurite [114]. The dose of dopamine we used was higher than those previously reported to elicit neurite retraction in neurons but our incubation time was shorter. Of note, there is data indicating that at the synaptic level, dopamine concentration can go beyond 1 mM [147].

We also investigated whether MDNCs conduct electrical activity. Twenty MDNCs coming from five individuals were tested. Three of those cells delivered inconclusive results while six cells did not show electrical activity. Over 200 recordings were obtained from the eleven remaining MDNCs. We encountered spontaneous action potentials (APs), excitatory postsynaptic potentials (EPSPs) and inhibitory postsynaptic potentials (IPSPs) [114]. The average membrane resting potential was −51 mV with a range of −34 to −65 mV while the average frequency for APs was 0.08 Hz with a range of 0.01 to 0.14 Hz and the average mean amplitude was 43.5 mV with a range of 37 to 50 mV. For EPSPs, the average frequency was 0.27 Hz with a range of 0.005 to 0.9 Hz and the average mean amplitude was 12.2 mV, whereas for IPSPs, the average frequency was 0.4 Hz with a range of 0.003 to 0.87 Hz and the average mean amplitude was −8.8 mV with a range of −20 to −2 mV. Those MDNCs that conducted electrical activity had a neuronal shape [114].

We used two approaches to establish differentiation efficiency for MDNCs. First, we relied on cellular phenotype. We considered differentiated MDNCs only cells with neuronal shape, meaning those cells with a well-defined soma and thin neurites longer than two times the soma size (Fig. 2), as it was in these cells where we found electrical activity, increased expression of neuronal markers, decrease expression of CD14 and a similar structural response to that of neurons when treated with colchicine and dopamine [114]. Characterizing 40,000 cells from 15 individuals via cellular phenotype, revealed a differentiation efficiency of 11.9 ± 1.4% [114]. Blood samples from these 15 individuals were collected in standard EDTA tubes. To determine if blood collection methods affect differentiation, we characterized another 5,000 cells from three individuals which blood was obtained via leucoreduction filters. This replication cohort showed a very similar differentiation efficiency of 12.8 ± 0.32% [114]. Our second approach to determine differentiation efficiency consisted of measuring expression of Postsynaptic Density protein-95 (PSD-95) via flow cytometry. PSD-95 a protein involved in synapse maturation [148, 149] was expressed in 36% of cells coming from eight individuals [114]. Based on these results we concluded that differentiation efficiency for MDNCs is between 11 to 36%, though the more stringent criteria suggests it is around 11%. This is a low differentiation efficiency when compared with some of the other cellular approaches described here (Table 1). Low differentiation efficiency could be problematic for certain scientific studies. The evidence currently available indicates that pluripotent monocytes are a subpopulation of circulating monocytes expressing CD14+CD34^low^ [123]. Methodologies that exclusively isolate CD14+CD34^low^ cells are likely to drastically increase the yield of cells amenable to differentiation. It is also important to mention that while obtaining MDNCs is a relatively rapid process as it only takes about 20 days, viability drastically decreases after day 25 of culture. We are currently testing several growth factors to maintain MDNCs in culture for longer periods of time.

One of the main challenges for IPSCs, iN cells and brain organoids is delivering reproducible results. [6–9, 100, 102, 103] Therefore, we investigated whether MDNCs results in serial samples from four healthy men remain consistent. Samples obtained 1.5 to 5 months apart showed reproducibility in differentiation efficiency and several neurostructural parameters including; number of primary, secondary and tertiary neurites as well as longest primary and secondary neurites [114]. Expression of dopamine 1 receptor (D1R) measured via flow cytometry was also consistent in all except one individual. For this individual, a third sample was collected which delivered comparable results to those encountered in the first sample [114].

### Psychiatric & neurological applications of non-genetic-transdifferentiation of monocytes

In 2012, pluripotent monocytes were tested as a potential treatment alternative in a rat model for cerebral ischemia [150]. Rats were subjected to occlusion of its left middle cerebral artery for one hour, which led to severe neurological deficits confirmed by magnetic resonance imaging of the brain. Three different treatment approaches were tested. All consisting of intracranial transplantation after a week of the ischemic event. One group of rats received syngeneic pluripotent monocytes, another syngeneic macrophages and the last group was transplanted with only cell culture media to serve as control. Pluripotent monocytes were generated using the method described by Kuwana et al. [116]. At baseline, neurological deficits were comparable between groups but after transplantation, rats that received pluripotent monocytes showed improvement when compared with the other two groups [150]. Such recovery was evident through the corner test, which is used to detect unilateral abnormalities of sensory and motor functions in rodent stroke models. Animals treated with pluripotent monocytes also evidenced a significant increase in vascularization around the ischemic area. By using pluripotent monocytes expressing green fluorescent protein (GFP), the authors showed that some of these cells differentiated into mature endothelial cells, though only 8% of these mature endothelial cells remained in situ four weeks after transplantation. The authors acknowledged that the underlying recovery mechanism remains unclear, but suggested it is likely related to angiogenesis promoted by pluripotent monocytes [150]. No differentiation into neuronal-like cells was observed.

The team who first differentiated pluripotent monocytes into retina-like cells also described how these cells integrated into mice’s retinas four weeks after intravitreal injection [134]. They later tested whether retina-like cells would behave the same in retinal degeneration slow (rds) mice, a model of recessive retinitis pigmentosa. Of note, retinitis pigmentosa is the most common genetic cause of blindness in humans and is characterized by loss of photoreceptors. Three months after subretinal transplantation in rds mice, retina-like cells were seen in the ciliary body, retinal outer nuclear layer, inner nuclear layer, ganglion cell layer, optic papilla and within the optic nerve [151]. However, despite the widespread location of retina-like cells, the structure of the retina did not change [151]. Six months after transplantation, retina-like cells expressing nestin, vimentin, MAP2, β3-tubulin or rhodopsin were still observed in the retina [135]. Also present, were cells derived from pluripotent monocytes that did not express any neuronal markers [135]. These promising results led the same team to test their transplantation method in retinal degeneration 1 (rd1) mice, which are considered a more severe model for retinitis pigmentosa. Thirty days after transplantation into rd1 mice, retina-like cells integrated into the retinal ganglion cell layer and the inner nuclear layer of the retina. These cells expressed nestin, MAP2 and the photoreceptor rhodopsin. But cell integration a month after transplantation was not sufficient to elicit electroretinographic waves [152]. In contrast, three months after transplantation, electroretinographic waves were recorded in all rd1 mice tested, while at five months, four out of nine eyes conducted electrical impulses [135]. Furthermore, cell integration and electrical activity were also present in the untreated eye [136]. These results suggest that transplanted retina-like cells migrate from the treated eye to the untreated one. How migration occurs is still under investigation [136]. The amplitude of electrical waves recorded in transplanted rd1 mice was not at the level of wild type mice. Therefore, the authors concluded that subretinal transplantation of retinal-like cells derived from human pluripotent monocytes provided a partial rescue of retinal function [135]. An independent research team obtained results consistent with this conclusion. As mentioned in a previous section, Mishra et al. developed their own protocol to transdifferentiate human pluripotent monocytes into what they termed retinal neuron-like cells (RNLCs) [127]. These RNLCs were transplanted into immunocompromised rd1 mice. At 10 days post transplantation, RNLCs were engrafted in the inner nuclear layer and the ganglion cell layer of the retina. Expression of several human specific retinal markers was evident [127]. Four weeks after transplantation, around 1.2% of RNLCs were still present. The impact of integrated cells was made evident by a combination of behavioral tests which indicated that depth perception in light and dim conditions improved in transplanted rd1 mice. Furthermore, exploratory behavior also increased in these animals but aversion to light was not impacted by treatment [127].

Monocytes have been used in different ways to further our understanding of psychiatric illnesses. For instance, monocytes physiological capacity to differentiate into microglia has been employed in vitro to explore potential etiological mechanisms in schizophrenia (SCZ) [153]. Several research teams have also assessed whether the number of circulating monocytes differs between patients with this psychotic disorder and healthy controls [154]. But to our knowledge, we are the first team to take advantage of monocytes’ capacity to transdifferentiate into neuronal-like cells to study the pathophysiology of SCZ. Our approach consisted on first, expanding the cohort in which we tested if MDNCs deliver reproducible results. We had originally published results from four men [114]. Another four individuals were added, one men and three women [146]. We also increased the number of samples in two subjects from two to three serial blood draws. We encountered differences in the percentage of differentiated cells but practically no differences in the number of MDNCs [146]. These results are consistent with previous literature suggesting there is a specific population of circulating monocytes with pluripotent capacity [123]. Our data indicates that the number of pluripotent monocytes remains constant with serial samples from the same individuals and thus, variations in the percentage of differentiated cells occur due to changes in the amount of other white blood cells but not pluripotent monocytes. Then, we tested the reproducibility of several neurostructural parameters. LPN and longest secondary neurite (LSN) remained consistent [146]. Number of primary neurites revealed inconsistencies while, number of secondary neurites and number of total neurites were reproducible [146].

Once we established which results were reproducible, we proceeded to compare MDNCs with HDNs in vitro. We had previously shown that the structure of HDNs after 5 days in culture was comparable to that of MDNCs [114]. We then compared the structural response to colchicine in these two cell types. Colchicine is known for its ability to retract neuronal extensions by directly depolymerizing microtubules and thus acting independently of membrane receptors [141, 155]. HDNs and MDNCs respond similarly to colchicine when LPN, number of primary neurites and total number of neurites are analyzed [146]. Secondary neurites however, respond differently. These results indicate that not only the neuropil of MDNCs and HDNs is comparable but also, that the similarities extend to how microtubules respond to colchicine. With this information at hand, we then moved on to contrast the structure of MDNCs from controls versus those from patients with SCZ. Twelve controls and 13 patients were included in the analysis. There were no differences in gender, age, number of PBMCs or number of monocytes between groups. But the percentage of monocytes was higher in patients which is consistent with previous results reported by several independent teams [154]. The transdifferentiation process was very similar between the two cohorts. In contrast, the percentage of differentiated cells determined by cellular phenotype was significantly higher in patients [146]. We assessed cell differentiation via two other means. One relied on expression of CD14, a marker of monocytes and macrophages. We have previously shown that CD14 decreases with neuronal transdifferentiation [114]. MDNCs from controls behave as expected, but cells from patients did not, as its expression of CD14 did not diminish [146]. Expression of nestin was also different between groups. MDNCs from patients expressed more nestin than cells from controls. Another important difference was the neurostructure. MDNCs from patients developed a more sophisticated structure, extending longer secondary neurites and growing more primary neurites than cells from controls [146].

Following, we challenged the neurostructure of MDNCs with three concentrations of colchicine (0.4, 0.5 & 0.75 µM) and two concentrations of dopamine (4 mM & 5 mM). After accounting for differences in differentiation efficiency and neurostructure at baseline, a mixed model analysis revealed no differences in the amount of retraction observed in MDNCs with any of the concentrations of colchicine tested [146]. The lower concentration of dopamine also exposed no differences between groups. However, the higher concentration of dopamine, elicited increased pruning of primary neurites in medicated patients with SCZ. Two of the 13 patients were not taking any medications. If these two patients were included in the analysis then differences in pruning were not observed [146]. Similarly, MDNCs from medicated patients expressed less D1R than cells from controls, but this difference was not evident when all patients were included in the analysis. These results suggest antipsychotics could be responsible for the differences between groups. However, several factors have to be considered before reaching such conclusion. Monocytes remain in circulation for 2 to 3 days [156] which would be the time they are exposed to antipsychotics. Our transdifferentiation protocol begins by isolating monocytes entirely from its circulating environment. Then, these cells are kept in culture for over 20 days. While in culture, media is replaced in four different occasions [114]. In addition, any antipsychotic effect would have to endure not only the prolonged culture period but also a drastic structural transformation that begins with rounded monocytes and ultimately delivers cells with complex shapes (Fig. 2). Furthermore, monocytes do not express dopamine receptors [146, 157]. Thus, decreased expression of D1R cannot be explained by antipsychotics interacting directly with this or any other dopamine receptors. Finally, we treated monocytes with blood circulating concentrations of haloperidol during days 4 to 7 of transdifferentiation in order to mimic the exposure time monocytes experience in circulation. Haloperidol did not elicit any changes in structure, pruning or expression of D1R in MDNCs [146]. However, the possibility remains that other antipsychotics may influence any of these parameters differently than haloperidol. In fact, there is evidence indicating that olanzapine [158] and risperidone [159] can decrease the expression of D1R by increasing DNA methylation. Consequently, our results showing decreased expression of D1R in medicated patients with SCZ could be explained by antipsychotic intake. This opens the possibility of a potential protective role of antipsychotics against dopamine-elicited pruning. Our results showed that D1R are partially responsible for dopamine pruning effects on LPN [146]. Since we found no differences in pruning of LPN between SCZ and controls after MDNCs were exposed to two different concentrations of dopamine, it is possible that by lowering D1R, antipsychotics prevented pruning of LPN in SCZ. In general, antipsychotics tend to decrease dopaminergic transmission, if so, then the impact of antipsychotics would have been to minimize rather than promote pruning. But how to explain the fact that we observed increased dopamine-elicited pruning only in medicated patients. We speculate that our results are due to patient heterogeneity, meaning that, not all patients are susceptible to the pruning effects of dopamine. In our cohort, the subset of patients susceptible to the pruning effects of dopamine, were those receiving antipsychotics. Perhaps, unmedicated individuals had a milder presentation of SCZ and thus, did not require continuous antipsychotic treatment, while medicated patients were severely ill and in need of treatment. Further research is clearly needed in order to elucidate the role of antipsychotics in dopamine-elicited pruning in MDNCs. A particularly promising approach to clarify the potential impact of antipsychotics, would be to examine the epigenetic landscape of MDNCs from patients taking medications and contrast them with both; unmedicated patients and healthy controls.

## Discussion

Many different research strategies are currently utilized to disentangle the complex pathophysiology of psychiatric illnesses, a quest that is far from complete. A noteworthy difference between research tools is its capacity to accommodate experimentation at a cellular level. Animal models are widely used to investigate potential cellular deficits. But rodents and non-human primates do not carry the genetic load that lead to mental illness. Moreover, their brains are clearly distinct from that of humans. It is therefore not surprising that translating discoveries from rodents to humans has not been fruitful within the psychiatric field. In contrast, a more productive methodology to investigate the role of neurons and glia in mental illness is the examination of postmortem tissue. Postmortem research in psychiatry has delivered many consistent results, among them, are indications of deficits at a cellular level [160, 161]. But working with dead tissue prevents the implementation of functional assays and consequently, studying physiological alterations in neurons and glia is unfeasible. Directly, acquiring neurons and glia from living patients would solve this problem. However, access to actual neurons is currently achieved almost exclusively through biopsies of the olfactory neuroepithelium which yield is erratic [5, 11] and consists only of olfactory sensory neurons [5]. Considering these circumstances, it is easier to appreciate the value of having access to neuronal-like cells coming directly from living patients and hence, carrying the genetic vulnerability to mental illness.

Here we compared five cellular approaches currently used in psychiatry to obtain neuronal-like cells, namely; ONCs, MSCs, Pluripotent Monocytes, IPSCs and iN cells. Brain Organoids mostly originate from IPSCs and thus, are derivative of a cellular approach. ESCs are not included in this discussion because they do not come directly from patients. Each of these cell-based approaches offers its own set of advantages and disadvantages (Table 1) that can be summarized as follows. ONCs give researchers the possibility to work with either ex vivo tissue or acquire neuroprogenitor cells for its expansion and differentiation [5, 46]. ONCs can be obtained relatively rapid and are the only source of human mature neurons [5]. But the use of ONCs has been limited due to accessibility, as a biopsy of the olfactory neuroepithelium is an invasive procedure that requires consultation with a specialist [11, 53] making it less practical and more expensive (Table 1). MSCs also involve a biopsy, but in this case obtained from the bone marrow [33, 36]. Its cost is also increased due to this surgical intervention (Table 1). Similarly to ONCs, MSCs can deliver differentiated neuronal-like cells rapidly. What distinguishes MSCs from the rest of stem cells, is its capacity to regulate the immune system, seemingly displaying anti-inflammatory properties [42]. Therefore, MSCs are almost exclusively used as potential treatment alternatives for a variety of illnesses [40, 41] including psychiatric disorders [39]. While pluripotent monocytes have also been tested as treatment alternatives, recipients of these cells were not humans. Instead, they were used in animal models of stroke and retinitis pigmentosa [127, 134, 150]. In humans, pluripotent monocytes are supporting the study of SCZ at a cellular level [146]. Neuronal-like cells derived from pluripotent monocytes are the most affordable approach and among the fastest methods to deliver differentiated cells (Table 1). In addition, access to pluripotent monocytes is non-invasive as it only requires a standard blood sample [114, 137]. The downside is that these neuronal-like cells are also among the least characterized and their differentiation efficiency with the protocols currently available is low [114, 126, 127, 134]. Moreover, neuronal-like cells derived from pluripotent monocytes remain at very early stages of neuronal differentiation [114, 137]. We have reported reproducible results in certain parameters of MDNCs [114, 146] but these data have yet to be replicated by an independent team. IPSCs are the cellular approach most widely used even though, it takes the longest in delivering differentiated cells, only surpassed by brain organoids (Table 1). IPSCs, iN cells and Brain Organoids are the techniques that deliver the largest variety of neuronal types and the more differentiated cells. In addition, IPSCs offer the best characterized neuronal-like cells, but this thorough characterization has also revealed that IPSCs do not deliver consistent results between cell lines from the same individual [6–9]. This confounder is also true for iN cells and Brain Organoids. [100, 102–104, 109] The three genetic methods to generate stem cells namely; IPSCs, iN cells and Brain Organoids are also the most expensive ones (Table 1), particularly Brain Organoids due to the extended culture time and the special equipment required to build them [100]. Brain Organoids however, are the only method in which the interaction of neuronal-like cells and glial-like cells can be studied in three dimensions. In order to fully appraise the value of each cellular approach, other factors need to be considered.

An important issue to consider when using cell-based methodologies is that psychiatric illnesses are, for the most part, polygenic disorders. Such disorders pose the challenge of patient heterogeneity, which can turn into type two errors due to lack of statistical power. Despite this caveat, several independent teams using various cellular approaches to psychiatric illnesses have reported differences even when contrasting small cohorts of 3 or 4 subjects per group [3, 162]. Small cohorts allow for the detection of statistical differences when the target of study is the neuronal structure as has been shown for years within the postmortem field [163]. The problem becomes more complex when genetic and epigenetic data is involved. This is particularly pronounced when using IPSCs [164]. Not only because the reprograming process can elicit genetic and epigenetic abnormalities, [10, 26, 65–67] but also because IPSCs are clones from one single cell [10]. In addition, studies involving IPSCs have relied on the statistical power provided by different cell lines coming from the same individual, instead of generating cell lines from different individuals [164]. Potential solutions to increase the statistical power when working with IPSCs have been proposed elsewhere [165]. Of note, cellular approaches that avoid the reprogramming process offer to be, at least in part, a better alternative (Table 1). These methodologies are likely to minimize or even completely circumvent genetic and epigenetic anomalies, though such results are yet to be proven. Cell-based techniques independent of reprograming are also less time-consuming (Table 1) and therefore, more individuals can be studied simultaneously which increases the statistical power. Moreover, these cellular approaches are not clones from one single cell and consequently they are more likely to produce generalizable findings.

Also of note is that while there are differences in the degree of neuronal differentiation that each of these cellular approaches deliver, all neuronal-like cells, regardless of the method used, remain relatively immature. Induced Neuronal cells retain the epigenetic age of its cell of origin which makes them better positioned to study psychiatric disorders with onset in or after adulthood [10]. But in general, all six cellular approaches are better suited to investigate disorders with neurodevelopmental origins such as autism and SCZ. This poses a particular challenge. What to use as reference point or gold standard for comparison? Using developing human neurons in culture, as we [114, 146] and others [108] have done, seems to us the best approach to determine whether neuronal-like cells reproduce the neuronal process to be studied. Once it is established that neuronal-like cells replicate aspects of human neuronal physiology in vitro, a more complex challenge emerges. Most of what we know about cellular abnormalities in psychiatric illnesses comes from postmortem research. But postmortem studies carry its own set of advantages and disadvantages. Among its most significant disadvantage is that more often than not, brains come from older patients, which likely reflect end stage diseases. With such consideration in mind, one has to wonder if it is reasonable to expect that results from cellular approaches will reflect data acquired from postmortem brains. Especially when we know that both methodologies likely reflect two different facets of the same illness. One at the very early stages and the other at the very end of it. Moreover, if we were ever to expect that cellular approaches will replicate findings from postmortem analyses, then we have to determine which postmortem results are truly diagnostic differences and which are confounding artifacts. Among the potential confounding factors affecting postmortem research are the clinical circumstances preceding dead. These agonal states have raised significant concerns as they are systematically ignored and can directly impact gene-expression patterns and mitochondrial enzymes in human postmortem brains [166, 167]. Other factors that can influence postmortem results are brain pH and postmortem interval (PMI). These two confounders are often well-controlled. However, storage time, which can affect the neuronal structure [168] usually receives less attention. Controlling for cause of death is also extremely difficult, but its omission can influence RNA quality, particularly in deaths associated with hypoxia [169]. Hypoxia related deaths can also lower immunostaining of microtubule associated protein 2 (MAP2) in human brains [170]. MAP2 has been the target of various studies in the SCZ field and results have been inconsistent [171–174]. Whether hypoxia related deaths influenced these results is yet to be clarified. One more significant confounder in postmortem research is the impact of medications. At best, antipsychotic intake is controlled by exposing rodents or non-human primates to these medications and then examining its posthumous effects. But as we have mentioned before, the brains of rodents and non-human primates are strikingly different from those of humans. Therefore, the value of such comparisons is uncertain. At worse, the confounding influence of years of medications intake in postmortem brains is barely mentioned. These various confounding factors evidence that discriminating between artifacts and differences driven by diagnosis in postmortem research is extremely challenging. That however, has not prevented the scientific field from making posthumous brain examinations a crucial piece of the puzzle to disentangle the pathophysiology of mental illness [175]. For instance, applying state of the art genetic tools to postmortem samples, recently helped localized genes associated with SCZ and autism to specific layers in the dorsolateral prefrontal cortex [176]. Even more impressive, are current postmortem results that juxtaposed SCZ risk genes to samples from different brain regions from healthy individuals at various developmental ages. Such analyses, allowed researchers to conclude that early prefrontal development is involved in the pathophysiology of SCZ [177]. A potentially transformative approach is the isolation of living neuroprogenitor cells [178] as well as actual living neurons from postmortem samples [179], though these techniques have not been extensively studied, its potential future applications are compelling. In the meantime, the six cellular approaches described here, are the puzzle piece that can provide a neurodevelopmental perspective but with its own set of complications, thus far unavoidable and equivalent to the current circumstances involving postmortem research. Among the confounders that plague cell-based methodologies are cell heterogeneity, including variability in stages of differentiation within the same cellular culture. There is also variability related to the use of different types of serums to complement cell culture media. In addition, the search for the “perfect neuron” has led to a myriad of differentiation protocols. Considering the difficulties to completely avoid confounding factors in postmortem research as well as in cell-based methodologies, establishing what results are meaningful for both approaches, will continue to depend on consistent and reproducible data obtained by independent research teams.

Once established that we have consistent and reproducible data obtained by independent research teams, is it then valid to question results coming from cellular approaches because it fails to align with postmortem findings? Let us use an example in which this was the case [164]. In patients with bipolar disorder, decreased neuronal-like cells viability in vitro [180] is allegedly [164] not consistent with postmortem results [161]. Indeed, a postmortem meta-analysis did not find widespread reductions in neuronal numbers [161]. But this report suggested that in some areas and for some neuronal types there could be a decrease in neuronal numbers in manic-depressive patients [161]. Moreover, the authors of such meta-analysis warned the reader not to reach any final conclusions due to the limited postmortem data currently available [161]. But even if the meta-analysis had definitively established that there were no changes in neuronal numbers in postmortem brains, a couple of circumstances have to be pondered. Firstly, the patients in which decreased neuronal-like cell viability in vitro was found are not the same individuals in whom postmortem studies were conducted. It remains to be established if those patients showing reduced neuronal-like cells viability in vitro, do present lower neuronal numbers in their brains. Secondly, it is unrealistic to expect that abnormalities occurring during early neuronal development will remain stable throughout life, and thus found in postmortem analyses. Physiological compensations in the face of illness and the effects of medications are only two of the innumerable factors that influence disease progression and ultimately alter early neurodevelopmental defects. If a cellular approach had to be selected to replicate postmortem findings, adult neurons obtained from the olfactory neuroepithelium are slightly better placed as they could be exposed to the innumerable factors that influence disease progression. Though, with the caveat that these adult neurons are in constant turnover [5, 46]. In general, postmortem findings which most likely represent end stage diseases are an inadequate reference point for cell-based methodologies which instead provide insights into neurodevelopmental processes. Therefore, invalidating cellular-based results because it fails to align with postmortem findings is misleading. Instead, what needs to be appreciated is that both types of information are valuable. On one hand, is the primary neurodevelopmental pathogenesis associated with mental disorders. While on the other, is the lifelong pathophysiology compensatory processes to overcome the primary pathology.

The example we used in the previous paragraph on decreased neuronal-like cells viability in vitro in patients with bipolar disorder [180] can also help us illustrate another potential scenario. If this finding were to become consistent and reproducible, even if only in a minority of bipolar patients, would such results be meaningful? With the research tools currently available, we cannot establish whether neuronal viability is decreased at some point during human brain development in patients with bipolar disorder. But this lack of validation does not mean such results are meaningless. There are several examples in medicine in which tests or compounds far removed from the original target become instrumental laboratory tools. For instance, creatinine levels are used as proxy of kidney function, even though creatinine is not part of the renal system [181]. Another example is the Venereal Disease Research Laboratory (VDRL) in use to diagnose syphilis. This test detects antibodies against antigens released by damaged cells but does not recognize the organism that causes the disease or antibodies against it [182]. The same principle applies for Rapid Plasma Reagin (RPR) [183] and both tests remain powerful tools in clinical practice despite the availability of more specific methods [182, 183]. Because they come directly from patients and thus carry the genetic predisposition to psychiatric illnesses, the six cell-based methodologies described in this manuscript carry the potential to serve as proxies. Though, some are more promising than others. The cost and time it takes to generate Brain Organoids makes this technique an unlikely alternative, at least presently. IPSCs are also constrained by time as the gap between recuperating somatic cells, reprograming and then differentiating neuronal-like cells is measured in months (Table 1). Another limitation with IPSCs is that the majority of studies using this technique include very small cohorts [164] that at times involve only one or two patients [184]. Cellular approaches that require a biopsy are also at a disadvantage (Table 1). Presently however, the main obstacle for all cellular approaches to psychiatric illnesses is to deliver consistent results that can be reproduced by independent laboratories. While none of the cell-based methodologies are even close to become a proxy, it is worth noting that research tools such as animal models or postmortem studies are completely voided of this possibility.

In conclusion, it is important to remember that our knowledge of mental illness in the realm of cells is, at best, scarce. Also important to consider, is that we do not need to understand a complete brain circuit or an entire brain region in order to pose valid scientific questions at a cellular level. We do need to ensure that neuronal-like cells coming directly from patients reproduce the cellular process to be studied, ideally using human neurons in culture as the gold standard. Regardless of the cellular approach to be used, confirming statistical power based on the number of patients and not only the number of cells is of particular importance. Just as relevant, is to establish that diagnostic differences are not driven by medication effects. But even after addressing all the manageable confounders, it cannot be forgotten that cellular approaches to psychiatric illnesses will remain imperfect research tools far removed from normal brain physiology and yet, it carries the potential to bring valuable scientific information and presumably, also the possibility to permeate clinical practice.
